# Drug-Coated Balloons: Recent Evidence and Upcoming Novelties

**DOI:** 10.3390/jcdd12050194

**Published:** 2025-05-20

**Authors:** Zaid Mohammad Fahmi Shahrori, Marco Frazzetto, Shamin Hayat Mahmud, Wasfi Alghwyeen, Bernardo Cortese

**Affiliations:** 1 Harrington Heart & Vascular Institute, University Hospitals Cleveland, Cleveland, OH 44106, USA; zaid.shahrori@uhhospitals.org (Z.M.F.S.); marcofrazzetto7@gmail.com (M.F.); shamin.mahmud@uhhospitals.org (S.H.M.); 2 DCB Academy, 20143 Milano, Italy; 3 Al-Nadeem Hospital, Ministry of Health, Amman 11118, Jordan; srajwasfi1@gmail.com; 4 Fondazione Ricerca e Innovazione Cardiovascolare, 26900 Milano, Italy

**Keywords:** DCB, drug-coated balloons, coronary artery disease, PCI

## Abstract

Drug-coated balloons (DCBs) have emerged as a compelling alternative to drug-eluting stents in the treatment of coronary artery disease (CAD), offering the advantage of local drug delivery without permanent vascular scaffold implantation. Initially developed for managing in-stent restenosis, DCBs seem appealing for broader indications, particularly in small vessel disease and bifurcation lesions. While paclitaxel-based DCBs remain the most investigated, newer limus formulations are showing promise and appear to be a valid alternative in early trials. Evidence from recent randomized clinical trials (RCTs) and meta-analyses highlights DCBs as a safe and effective option in selected patients, with potential benefits including lower restenosis rates, reduced need for dual antiplatelet therapy, and avoidance of late stent-related complications. As new large-scale trials near completion, DCBs are poised to take on a broader role in the treatment of CAD, particularly in patients where “leaving nothing behind” offers a clinical advantage. This review offers an overview of the DCB platforms commercially available, showing pharmacological differences, providing current indications in practical guidelines, and analyzing the most recent and impactful RCTs and meta-analyses in the field.

## 1. Introduction

Historically, the evolution of coronary artery disease (CAD) treatment aimed to improve outcomes and overcome the challenges of the existing standard by developing new technologies. The first percutaneous coronary intervention (PCI) utilized plain old balloon angioplasty (POBA), starting a revolution in the management of CAD [1]. POBA increased the luminal diameter by direct plaque compression and dilatation, but was often complicated by acute vessel closure due to elastic recoil [1].

Soon after, bare-metal stents (BMSs) were introduced to overcome the key pitfalls of POBA; however, BMSs were complicated by a high rate of in-stent restenosis (ISR) (15–35%), primarily caused by neointimal hyperplasia arising from inflammatory responses due to prolonged exposure to permanent metallic scaffolds [2]. Subsequently, first- and second-generation drug-eluting stents (DESs) reduced ISR rates by delivering antiproliferative drugs to the vessel, helping suppress neointimal hyperplasia. Nevertheless, ISR still occurred at a rate of 1–2% per year even in the latest DES iterations, and treatment for ISR accounted for 10% of all PCIs in the United States over the last decade [1,3]. Thus, despite recent advances in CAD treatment, complications such as ISR, late stent thrombosis (which carries high mortality rates), and bleeding risks associated with dual antiplatelet therapy (DAPT) create opportunities for alternative technologies.

## 2. Overview and Types of Drug-Coated Balloons

Drug-coated balloon (DCB) development, beginning in the early 2000s, initially focused on ISR management. DCB angioplasty involves coating a traditional balloon with an antiproliferative drug, which is then rapidly transferred directly to the arterial wall during balloon inflation, allowing for a stent-less form of PCI while suppressing neointimal hyperplasia [4].

DCBs deliver antiproliferative medications directly to the lining of the arterial wall. To achieve this, adequate lesion preparation is paramount to obtaining satisfying short- and long-term results with such technology.

Furthermore, several coating preparations to facilitate drug delivery have been developed for use in DCBs, with two major antiproliferative drugs, paclitaxel and sirolimus, being the most widely used. For effective drug transfer to the vascular wall, the coating preparations include a hydrophilic matrix excipient mixed with the drugs. The excipient, an inactive and non-reactive substance, enhances both the adherence of the drug coating to the vascular wall and the subsequent solubility of the drugs within the arterial lining, allowing them to dissolve and exert their effects on the vascular wall rapidly. Thus, the type and characteristics of the coating solution, including the choice of antiproliferative drug, directly determine drug transfer and long-term vascular effects [5].

**Figure 1:**
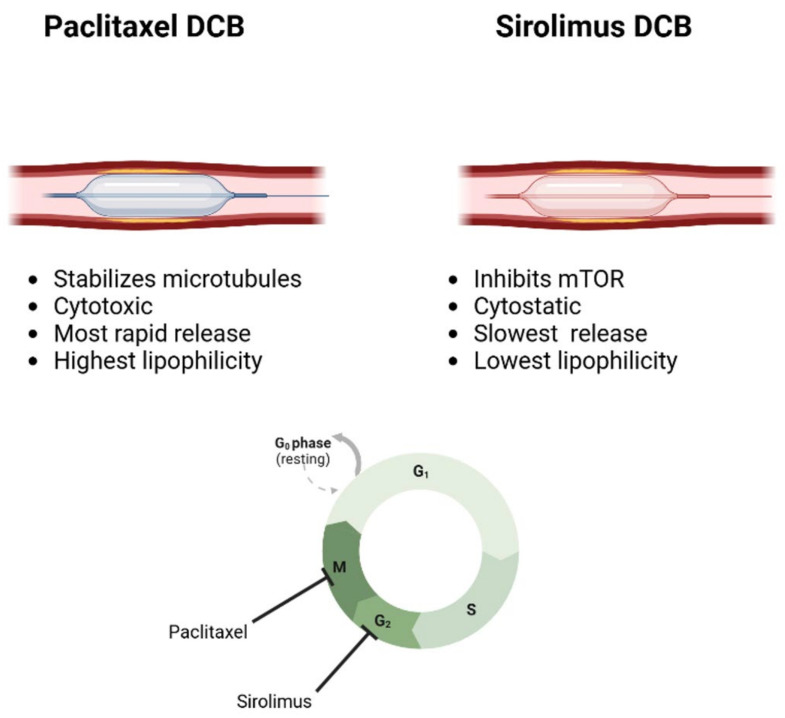
Different mechanisms of action for the drugs released by the DCBs.

Paclitaxel was the first and remains the most commonly used antiproliferative drug for DCBs. Paclitaxel inhibits the cell cycle by causing M-phase arrest, reducing the proliferation of smooth muscle and endothelial cells in the coronary arteries. Its use in DCBs is ideal for its rapid onset of action and solubility in the vessel, as well as its potent inhibition of microtubules and the consequent reduction in arterial neointimal hyperplasia [6] (Figure 1).

Sirolimus, an antiproliferative drug widely used in DES, has been explored in DCBs. Sirolimus acts by inhibiting mTOR, a pro-proliferative cellular kinase, which subsequently leads to S-phase arrest and reduces cell proliferation. Compared to paclitaxel, sirolimus has been associated with poorer penetration into the arterial wall, longer time to reach therapeutic levels, and shorter duration of action during DCB use, posing some challenges. Despite such limitations, coating preparations such as the MagicTouch SCB (Concept Medical), which uses nanoparticles as the excipient, can facilitate sirolimus penetration into the wall, rapid cellular uptake, and prolonged drug response [4] (Figure 1).

Table 1 lists an updated list of commercially available DCB technologies for use in CAD.

## 3. Current Applications for Drug-Coated Balloons

The role of DCBs in treating ISR has since been well-established, receiving a Class I A recommendation in the 2018 ESC/EACTS Guidelines, similar to DESs [7]. Recently, the 2024 ESC/EACTS Guidelines reiterated that DCBs are an option for ISR treatment, although the implantation of a second DES should be preferred for treating DES-associated ISR (Class I) [8].

These recommendations were based on the results of two recent meta-analyses. The first one compared the efficacy and safety of angioplasty with DCBs and repeat stenting DES—in patients with BMS and DES-ISR—showing both were equally effective in treating ISR, although DCBs were less effective than repeat DESs in treating ISR [9]. The second one demonstrated DCBs to be less effective than everolimus DESs in ISR management [10]. Notably, different authors have challenged this decision, stating that there is no recent and robust evidence to justify downgrading DCBs compared to DESs [11].

In the 2021 ACC/AHA/SCAI Guidelines, DCBs are recognized as a treatment option for ISR. However, American guidelines recommend that repeat PCI with a DES is preferred and provides the most benefit, being associated with improved rates of target vessel revascularization (TVR) and lower ISR rates [12]. Thus, ongoing research continues to shape our understanding of the exact role of DCBs in this context.

Beyond ISR management, DCBs showed encouraging results in de novo CAD lesions, especially in small vessel disease (SVD). DCBs could have a potential role in regions of DES pitfalls, as the latter tended to have suboptimal outcomes in small vessels and diffuse CAD [13]. In the treatment of de novo CAD, the literature has shown varying utility for DCB use compared to the latest generations of DES. A meta-analysis reported DCBs to have similar efficacies to DESs in the treatment of de novo coronary lesions and SVD, although, in the recent REC-CAGEFREE I trial, DCBs did not reach non-inferiority compared to DESs in low-risk de novo coronary lesions [10,14]. Nonetheless, DCBs remain a promising alternative, as they do not require permanent stent placement, theoretically mitigating the risk of ISR and delayed stent thrombosis seen with DES, while potentially having shorter DAPT durations [6].

## 4. New Randomized Controlled Trials and Meta-Analysis

Table 2 summarizes the novel trials and meta-analyses focused on DCB angioplasty in CAD.

### 4.1. REC-CAGEFREE I Trial [14]

The REC-CAGEFREE I trial was a non-inferiority trial for DCB angioplasty with rescue stenting vs. DES implantation in patients requiring PCI for de novo, non-complex coronary lesions. Conducted at 43 centers in China, the trial included 2272 patients aged 18 or older, with follow-up extending to 24 months. The trial population primarily consisted of patients with preserved left ventricular function, low rates of prior myocardial infarction or PCI, and non-complex coronary lesions, reflecting a low-to-moderate cardiovascular risk profile. The devices used were the Swide DCB (Shenqi Medical) and the Firebird 2 DES (MicroPort), both of which are widely utilized in China.

The primary outcome was the device-oriented composite endpoint (DoCE, including cardiovascular death, target vessel myocardial infarction, and clinically and physiologically indicated target lesion revascularization [CPI]). The study failed to prove the non-inferiority of DCB-PCI (*p*_non-inferiority_ = 0.65). The primary endpoint, the device-oriented composite endpoint (DoCE), occurred in 6.4% of patients in the DCB group and 3.4% of patients in the DES group.

The study found that rates of cardiovascular death and target vessel myocardial infarction (TV-MI) were similar between the DCB and DES groups (2.3% vs. 1.2%, *p* = 0.054; 1.9% vs. 1.6%, *p* = 0.61, respectively). However, DESs were associated with significantly lower rates of clinically indicated target lesion revascularization (CPI-TLR) and overall revascularization (3.1% vs. 1.2%, *p* = 0.0021; 7.0% vs. 4.6%, *p* = 0.016, respectively). Major bleeding events were numerically less frequent in the DCB group (1.4% vs. 2.4%, *p* = 0.096), potentially due to earlier cessation of DAPT. Additionally, DCB patients experienced a higher incidence of net adverse clinical events (12.2% vs. 9.2%, *p* = 0.022), defined as a composite of the patient-oriented composite endpoint (PoCE) and BARC-defined type 3 or 5 bleeding events. The PoCE itself included all-cause death, any stroke, any myocardial infarction, and any revascularization.

Importantly, a subgroup analysis revealed comparable outcomes between the two strategies in SVD (vessel diameter < 3.0 mm), with the primary composite endpoint occurring in 5.1% of DCB patients vs. 4.4% of DES patients (*p* = 0.59). These findings suggest that DCBs may offer a viable alternative to DESs in certain populations, particularly those at risk of bleeding, adverse stent-related complications, and patients with SVD. The failure to demonstrate non-inferiority was attributed to the higher rates of revascularization in the DCB group, which may reflect inherent limitations of DCBs (in some settings) in achieving durable outcomes compared to DES. The inability to achieve non-inferiority emphasizes the necessity for rigorous patient selection in DCB applications, such as lesions in SVD.

### 4.2. Dissolve SVD [13]

The Dissolve SVD is a prospective, randomized, multicenter, non-inferiority trial comparing Dissolve DCB (DK medical) against the Resolute Onyx DES (Medtronic) in the treatment of single lesion de novo small vessel CAD.

The Dissolve DCB is a next-generation device coated with 3 mg of paclitaxel/mm^2^ using a midchain triglyceride excipient, which enhances drug retention and rapid penetration into the vascular wall compared to earlier DCBs.

The cardiovascular risk profile of the patients was categorized as low to moderate. Key inclusion criteria were reference vessel diameters between 2.00–2.75 mm, lesion lengths of <26 mm, and a target lesion stenosis of ≥70% or ≥50% with documented myocardial ischemia. Major exclusion criteria included acute myocardial infarction within one week, severe heart failure (NYHA Class IV), extensive thrombus, bifurcation lesions requiring side-branch intervention, and severe renal impairment (GFR < 30 mL/min). These criteria ensured a focus on relatively straightforward and stable cases, reducing the overall risk profile of the enrolled population.

The trial included 247 patients treated in 10 Chinese centers. An additional cohort of 30 patients with very small vessels (RVDs ≥ 2.00 and <2.25 mm) was enrolled. The primary endpoint was in-segment percentage diameter stenosis at 9 months. The results demonstrated the non-inferiority of the Dissolve DCB compared to the Resolute DES (31.2% ± 2.0% vs. 26.1% ± 2.1%, *p*_non-inferiority_ = 0.0002).

Late lumen loss (LLL) favored the DCB group (0.22 ± 0.35 mm vs. 0.31 ± 0.38 mm, *p* = 0.09), though it was not statistically significant.

At 12 months, target lesion failure (8.5% vs. 6.1%, *p* = 0.28) and major adverse cardiovascular and cerebrovascular events (20.9% vs. 13.6%, *p* = 0.12) were comparable. Furthermore, this study showed the very limited need for bailout stenting, 3.9%, which could be attributed to the mandatory pre-dilation.

### 4.3. ANDROMEDA Patient-Level Meta-Analysis [15]

A recent patient-level meta-analysis, including 1475 patients from four randomized clinical trials (RCTs), investigated the 3-year outcomes of PCBs vs. DESs in de-novo SVD-CAD. A cut-off of ≤3 mm was chosen to define a small vessel. The results showed that PCB is associated with a statistically significant lower risk of major adverse cardiac events (MACEs), a composite of all-cause death, myocardial infarction, and/or TVR, compared to DESs (18.5% vs. 24.5%, respectively), remaining consistent after multivariable adjustment. Lower risk for MI and TVR drove the primary endpoint. Additionally, target lesion thrombosis was also significantly reduced in the PCB group compared with the DES group (HR 0.20, 95% CI 0.04–0.96, *p* = 0.044). The co-primary endpoint was target lesion failure (TLF), a composite of cardiac death, myocardial infarction, or target lesion revascularization, and did not differ between groups (14.7% vs. 17.6%, respectively). These findings, derived from the entire body of the strongest evidence in the field, suggest that PCB angioplasty offers MACE reduction at 3 years, showing a preferential benefit of PCBs in small vessel disease over DESs and confirming the central role of DCB in the treatment of small vessels. To date, the Andromeda study is the best evidence available for the overall assessment of the DCB performance in small vessels, and it has the potential to introduce, in the future, practical guidelines for the use of DCBs in SVD.

### 4.4. Transform I [16]

The TRANSFORM I study was a randomized non-inferiority trial comparing the sirolimus-coated balloon MagicTouch vs. the paclitaxel-coated balloon SeQuent Please Neo (B. Braun) in treating de novo SVD. Conducted in multiple European centers, the randomized trial included 121 patients with stabilized acute or chronic coronary syndromes and small coronary vessel lesions, with an average reference vessel diameter (RVD) of 2.05 mm. The primary endpoint for non-inferiority was angiographic net lumen gain at six months, and the cut-off was set at 0.30 mm. The SCB failed to demonstrate non-inferiority, achieving a mean angiographic net gain of 0.25 ± 0.40 mm compared to 0.48 ± 0.37 mm for the PCB (absolute difference: −0.23 mm; 95% CI: −0.37 to −0.09 [*p*_non-inferiority_ = 0·173]). This result was primarily driven by the significantly smaller late lumen loss observed with the paclitaxel-coated balloon (0.00 ± 0.32 mm vs. 0.32 ± 0.47 mm; *p* < 0.001) and its higher incidence of late lumen enlargement (53.7% vs. 30.0%; OR: 2.60, 95% CI: 1.22–5.67; *p* = 0.014). Additionally, the correlation between late luminal loss and acute dissection volume, as measured by optical coherence tomography, indicates that the MagicTouch coating does not effectively prevent restenosis induced by barotrauma. Nevertheless, this is a small study and does not diminish the potential of the technology overall, which will be fully assessed in the future via a powered study for clinical endpoints. Moreover, lesion preparation was deemed inadequate in some centers, favoring paclitaxel, which is a more lipophilic drug [17].

### 4.5. SeQuent SCB vs. SeQuent PCB [18]

Another notable non-inferiority trial assessed a sirolimus-coated balloon SeQuent SCB (B. Braun) with a crystalline coating against a paclitaxel-coated balloon SeQuent Please Neo for treating de novo coronary lesions. Conducted at four centers in Germany and Switzerland, this randomized trial included 70 patients with stable and unstable angina and de novo stenosis and focused on LLL at six months as the primary endpoint for non-inferiority. The mean reference vessel diameter was 2.95 mm. The SCB group demonstrated non-inferiority to the PCB group, with in-segment LLL of 0.11 ± 0.37 mm vs. 0.04 ± 0.39 mm (mean difference: 0.07 mm; 95% CI: −0.12 to 0.26). The prespecified non-inferiority margin was high, set at 0.35 mm [18].

The conflicting results underscore the need for large randomized trials to understand the potential role of SCB in clinical practice and the overall class effect of DCB in the treatment of de novo CAD.

### 4.6. ISAR-DESIRE 3A, 7-Year Outcomes [19]

This study examined the 7-year outcomes of the control arm of the original ISAR-DESIRE 3 trial. It evaluated two PCBs for managing DES-ISR: Agent PCB (Boston Scientific), featuring a low-dose citrate excipient, and SeQuent Please PCB, with a standard iopromide excipient. A total of 262 patients and 323 lesions were enrolled. Long-term outcomes, including rates of target lesion revascularization (TLR), death, MI, and target lesion thrombosis (TLT), were examined.

At the 7-year follow-up, no statistically significant differences were found between Agent and SeQuent Please in terms of target lesion revascularization (43.2% vs. 35.9%; HR 1.29, 95% CI 0.87–1.90; *p* = 0.205), death (26.8% vs. 20.2%; HR 1.38, 95% CI 0.82–2.35; *p* = 0.227), cardiac death (18.2% vs. 14.0%; HR 1.42, 95% CI 0.75–2.67; *p* = 0.279), myocardial infarction (5.9% vs. 5.2%; HR 1.10, 95% CI 0.39–3.15; *p* = 0.852), or target lesion thrombosis (1.6% vs. 0.7%; HR 2.18, 95% CI 0.20–24.10; *p* = 0.523). Both PCBs provided comparable efficacy and safety for long-term DES-ISR management, reinforcing their role as effective treatment options.

### 4.7. ISAR-DESIRE 3, 10-Year Outcomes [20]

The ISAR-DESIRE 3 trial was a randomized, open-label comparative study aimed at assessing the long-term efficacy and safety of three strategies for the treatment of DES-ISR: plain balloon angioplasty (PB), paclitaxel-coated balloon angioplasty (PCB, SeQuent Please), and paclitaxel-eluting stent implantation (PES, Taxus Liberté, Boston Scientific, Mascot, NSW, Australia). The study included 402 patients with 500 lesions.

The primary outcome was a device-oriented composite of cardiac death, target vessel myocardial infarction (MI), target lesion thrombosis (TLT), or target lesion revascularization (TLR). At 10 years, the primary endpoint occurred in 72.0% of PB, 55.9% of PCB, and 62.4% of PES groups (*p* < 0.001). Pairwise comparisons showed no significant differences between PCBs and PESs (HR: 1.10, 95% CI: 0.79–1.52, *p* = 0.610).

Secondary outcomes included TLR and major safety events (cardiac death, target vessel MI, or TLT). TLR rates were significantly lower in the PCB (43.9%) and PES (38.6%) groups compared to PB (58.0%, *p* < 0.0001). While no significant differences were noted between PCBs and PESs for safety endpoints (HR: 1.26, 95% CI: 0.82–1.95, *p* = 0.564), even if a numerically higher rate of cardiac deaths was observed in PESs during the first five years (HR: 2.59, 95% CI: 1.07–6.30, *p* = 0.047), this difference attenuated over 10 years. This trial is significant for providing the longest follow-up data (10 years) for DES-ISR management strategies, highlighting comparable efficacy and safety between PCBs and PES.

### 4.8. BIO ASCEND ISR [21]

The BIO ASCEND ISR trial evaluated the non-inferiority of a novel biolimus-coated balloon (BCB) (BiolimusAscend [JW Medical Systems]) against an established PCB (SeQuent Please NEO) for treating DES-ISR. Biolimus (BA9, Biosensor International, Singapore) is a sirolimus analog modified to increase lipophilicity.

In a previous study, a BCB demonstrated superior outcomes compared to PBs in small-vessel CAD [22]. This randomized, multicenter trial included 280 patients across 17 centers, with LLL at 9 months as the primary endpoint, and a non-inferiority margin of 0.195 mm. The non-inferiority was met, with LLL values of 0.23 ± 0.37 mm for BCB and 0.25 ± 0.35 mm for PCB (mean difference: −0.02 mm; 95% CI: −0.12 to 0.07 mm; *p* < 0.0001 for non-inferiority). A prespecified optical coherence tomography (OCT) substudy confirmed similar neointimal areas between groups. Clinical outcomes at 12 months were also comparable, even if TVR was numerically higher in the BCB group (17.0% vs. 9.6%, *p* = 0.068). These findings highlight the potential of BCBs as a safe and effective alternative for managing DES-ISR, with comparable angiographic and clinical outcomes to those of the paclitaxel-coated balloon; however, the results from this trial have not been confirmed by the REFORM study, calling into doubt the efficacy of such technology.

### 4.9. REFORM [23]

The REFORM study investigated the non-inferiority of the biolimus A9-coated balloon compared to the SeQuent Please PCB in the treatment of ISR. Conducted across 20 centers in six countries, the study randomized 202 patients in a 2:1 ratio to BCB or PCB treatment. The primary endpoint, in-segment percentage diameter stenosis at six months, was significantly higher in the BCB group (43.3% ± 22.9%) compared to the PCB group (31.4% ± 17.7%), failing to demonstrate non-inferiority (*p* = 0.48). Secondary angiographic outcomes, including late lumen loss and binary restenosis, also favored the PCB group. At one-year follow-up, there were no significant differences in target lesion failure (23.7% in BCB vs. 17.1% in PCB, *p* = 0.28). Despite a similar design and comparable lesion complexity, calcific burden, ISR lesion type, and the pre-procedure minimal luminal diameter of the BIO ASCEND ISR trial, this study failed to demonstrate non-inferiority based on angiographic outcomes at 6 months. Interestingly, patients in the BCB group underwent more aggressive plaque preparation, yet this did not result in a higher rate of procedural complications. Additionally, the dissection rate was lower in the BCB group (0.7%) compared to the PCB group (7.5%; *p* = 0.056). This is paradoxical, as more intensive lesion preparation should, in theory, enhance drug delivery to vascular tissues and improve long-term antiproliferative effects.

The primary reason for the results lies in the challenges associated with efficacy drug transfer and tissue retention using the biolimus-coated balloon, emphasizing the need for further development before such a balloon can be evaluated in large studies or considered a viable alternative to established PCB technologies.

### 4.10. AGENT IDE [24]

The AGENT IDE trial was a multicenter, randomized, single-blind study comparing the efficacy and safety of the Agent PCB vs. an uncoated balloon for treating coronary ISR.

The AGENT IDE trial was conducted to fill a critical gap in the treatment of coronary ISR within the United States, where DCBs had not yet been approved for treating ISR. This research builds on earlier international studies, including those from a decade ago, which compared the effectiveness of PCBs with uncoated options for treating ISR [25,26]. The trial focused on the application of DCB technology in specific lesion types, particularly those with reference vessel diameters of 2.0–4.0 mm and lengths shorter than 26 mm.

The trial enrolled 600 patients across 40 centers in the United States. The primary endpoint was 1-year (TLF), a composite of ischemia-driven target lesion revascularization, target vessel-related myocardial infarction, or cardiac death. The PCB group demonstrated significantly lower rates of TLF at 17.9% compared to 28.6% in the uncoated balloon group (HR, 0.59; 95% CI, 0.42–0.84; *p* = 0.003). Additionally, ischemia-driven target lesion revascularization (13.0% vs. 24.7%; HR, 0.50; *p* = 0.001) and target vessel myocardial infarction (5.8% vs. 11.1%; HR, 0.51; *p* = 0.02) were reduced with PCB treatment. There was no significant difference in cardiac death rates between the groups (2.9% vs. 1.6%; *p* = 0.38). The trial also reported no stent thrombosis in the PCB group compared to six cases (3.2%) in the uncoated group.

Recently, at the 2025 CRT Conference, the 2-year outcomes were presented. Agent DCB confirmed superior outcomes compared to BA in terms of TLF (34% vs. 27%, respectively) and TVR (29% vs. 20%, respectively). The study confirmed once again that DCBs outperformed uncoated balloons in ISR, and finally, it led to the adoption of the technology in the United States.

### 4.11. DCB-BIF [27]

This multicenter, randomized trial evaluated the efficacy of a polymer-free PCB (SeQuent Please NEO) vs. noncompliant balloons (NCBs) (Sprinter Legend [Medtronic]) for treating pinched side branches after provisional stenting in patients with simple, true coronary bifurcations. The study enrolled 784 patients across 22 centers in China, Indonesia, Italy, and Korea with true coronary bifurcations with a side branch diameter stenosis of greater than 70% after main vessel stenting. The primary endpoint was MACE at 1-year follow-up, defined as a composite of cardiac death, target vessel myocardial infarction, and clinically driven target lesion revascularization. The secondary endpoints included all-cause death, cardiac death, periprocedural and spontaneous myocardial infarction, angiographic success, procedural success, and stent thrombosis. The results indicated a significantly lower rate of MACE in the DCB group (7.2% vs. 12.5%; HR: 0.56; *p* = 0.013), predominantly due to a reduction in myocardial infarction rates (1.0% vs. 3.6%; HR: 0.27; *p* = 0.029). Both groups demonstrated comparable rates of secondary outcomes, such as stent thrombosis and all-cause mortality. This trial highlights the potential role of DCBs as an effective strategy for the treatment of side branches after provisional stenting in bifurcation lesions, reaffirming the results of previous trials such as PEPCAD-BIF [28]. The importance of this study is paramount because, in a modern PCI approach, most of the coronary bifurcations are treated with a provisional stenting technique, and the universal implementation of a DCB-based side branch treatment could further improve clinical outcomes. However, several technical issues related to the technique of bifurcation angioplasty should be underscored [29].

### 4.12. ULTIMATE III [30]

The ULTIMATE III trial was a prospective, multicenter, randomized controlled study that evaluated the efficacy of intravascular ultrasound (IVUS)-guided vs. angiography-guided DCB (SeQuent Please) angioplasty for de novo coronary lesions. The trial enrolled 260 patients from four centers in China, with a reference vessel diameter of 2.0–4.0 mm and lesion length of ≤15 mm in an all-comer population. The primary endpoint was in-segment LLL at seven months, which was significantly lower in the IVUS-guided group (−0.10 ± 0.34 mm) compared to the angiography-guided group (0.03 ± 0.52 mm; mean difference: 0.14 mm; *p* = 0.025). Secondary outcomes included target vessel failure (TVF) at six months, which showed a non-significant trend favoring IVUS guidance (0.8% vs. 3.1%; *p* = 0.370). Procedural metrics such as post-procedure MLD were larger in the IVUS group (2.06 ± 0.62 mm vs. 1.75 ± 0.63 mm; *p* < 0.001).

This trial demonstrated that IVUS-guided DCB angioplasty improves late lumen outcomes and procedural efficacy compared to angiography guidance. These findings support the use of IVUS in optimizing DCB outcomes; however, some considerations regarding the inclusion criteria should be underscored, such as the inclusion of lesions with length ≤ 15 mm, which contrasts with the usual practice of using DCBs in long lesions and in diffuse disease, where IVUS offers advantages over angiography.

### 4.13. PICCOLETO VI [31]

The PICCOLETO VI trial was an international, multicenter, non-inferiority study comparing the angiographic and functional outcomes of PCBs vs. SCBs in treating de novo small-vessel CAD. All patients underwent independent angiographic and physiological assessment by the core laboratory, including Murray’s law-based quantitative flow ratio (μFR). The primary endpoint was late functional loss. The study included 293 patients treated using either PCB (n = 148: Elutax III n = 34, Prevail n = 28, Sequent Please Neo n = 68, Pantera Lux n = 9, Restore n = 5, and Agent n = 4) or SCBs (n = 79: Magic Touch n = 51, Selution n = 25, and Sequent SCB n = 3).

PCBs showed lower late lumen loss (−0.05 ± 0.56 vs. 0.10 ± 0.59 mm; *p* = 0.05) and a higher prevalence of late lumen enlargement (58.1% vs. 40.5%; *p* = 0.01). However, the primary endpoint of late functional loss was not statistically different between PCBs and SCBs (−0.01 ± 0.15 vs. +0.03 ± 0.13; *p* = 0.09). There was no difference in terms of target-lesion failure as well. A post-DCB μFR of ≤ 0.86 emerged as a reliable cut-off for predicting follow-up ischemia, with an accuracy of 80%.

In this direct comparison between several types of DCB, late functional loss was comparable between PCBs and SCBs, with PCBs confirming superior angiographic performance at 6 months. It is well known that PCBs offer better angiographic results at follow-up, but whether this leads to a reduction in clinical endpoints is debated. In consideration of the central role played by the coronary physiology assessment in the modern PCI, this study has an important role, as it can serve as a hypothesis generator for the physiological, rather than purely angiographic, assessment of the results obtained using different DCBs.

## 5. Future Perspectives

The diffusion of DCB angioplasty for the treatment of CAD will likely see a steady rise in the near future. Despite some controversy regarding DCB use in ISR patients due to recent changes in the European guidelines, randomized trials indicate that both DCB angioplasty and repeat stenting are viable clinical options.

The most rapidly advancing field of research focuses on the use of DCB angioplasty in de novo CAD, offering the clear advantage of treating coronary artery stenosis while avoiding scaffold implantation. The most appealing application of the DCB technology in de novo CAD seems related to the SVD and the side branch treatment in true bifurcations, considering the recent evidence [15,27]. However, the absence of high-quality, large-scale randomized clinical trial data prevents widespread adoption and guideline endorsement. Several RCTs are investigating different indications and CAD patterns for DCB angioplasty, with a significant proportion of these trials investigating the use of DCBs in de novo CAD (Table 3). There are two pivotal studies that are still ongoing: the SELUTION de novo trial is an RCT designed to compare Selution (Cordis, Santa Clara, CA, USA) SCBs with DES implantation in patients with de novo CAD, with randomization occurring before lesion preparation, and covering reference vessel diameters from 2.0 mm to 5.0 mm. With an enrollment target exceeding 3000 patients, it will be an important trial and will provide crucial insights into the future role of DCB angioplasty in de novo CAD.

TRANSFORM II is the second large-scale key RCT that aims to assess the non-inferiority of MagicTouch SCBs with the latest-generation DESs in the treatment of native coronary vessels with a diameter between 2 and 3 mm. The primary endpoint, tested for non-inferiority, is TLF, and the co-primary, tested for superiority, is the rate of net adverse cardiovascular events, both assessed at 12 months. Optimal lesion preparation is encouraged for all patients enrolled, especially in patients undergoing PCI with DCBs. If this condition is not met, the patient will be excluded from the study and followed up as a part of a nested registry [32]. With a goal of more than 1800 patients, this trial will further clarify the impact of DCB angioplasty in native vessel CAD.

## 6. Mechanistic Insights into DCB Performance

Beyond the device platform itself, the clinical performance of DCBs is significantly influenced by the characteristics of the drug and the excipient used in the coating formulation. The drug affects pharmacokinetics, including vessel wall uptake, retention, and antiproliferative action, while the excipient modulates drug release kinetics, bioavailability, and tissue diffusion. Understanding the interplay between drug properties and excipient behavior is essential to optimizing DCB performance across lesion types and vascular beds.

Paclitaxel remains the most extensively studied and commonly used antiproliferative drug in coronary DCBs. Its high lipophilicity and strong microtubule-binding properties make it well-suited for rapid tissue absorption during brief balloon inflation times, providing potent inhibition of neointimal hyperplasia. Conversely, sirolimus offers a more favorable vascular healing profile through the inhibition of the mTOR pathway but has historically demonstrated less efficient penetration into the arterial wall due to its hydrophilic nature. To address this, next-generation SCBs utilize novel excipient platforms such as lipid nanospheres, nanoparticles, or biodegradable polymers to facilitate drug uptake and prolong local tissue retention. Devices like the MagicTouch SCB and Selution SCB exemplify this approach, and several trials have examined their comparative performance with PCBs, with mixed results [14,31].

A third emerging antiproliferative agent in DCB development is biolimus, a sirolimus analog with enhanced lipophilicity. Biolimus is intended to combine the deep tissue penetration of paclitaxel with the favorable healing characteristics of sirolimus. Although initial results with biolimus-coated balloons (BCBs), such as in the BIO ASCEND ISR trial, suggested angiographic and clinical outcomes comparable to established PCBs, these findings were not replicated in the REFORM study, which failed to demonstrate non-inferiority of BCBs in key angiographic endpoints [21,23]. These mixed results underscore the complexity of achieving optimal drug release, transfer, and tissue effect, even with pharmacologically modified analogs like biolimus. The disappointing results from REFORM, despite the use of more aggressive lesion preparation in the BCB group, highlight the challenges in translating theoretical pharmacological advantages into clinical efficacy without refined excipient design and delivery control.

In summary, the combination of drug characteristics—such as lipophilicity, mechanism of action, and metabolic stability—and excipient features—such as hydrophilicity, matrix degradation profile, and molecular architecture—critically determines the efficiency and consistency of drug delivery in DCB systems. Future development must focus not only on identifying optimal drug candidates but also on engineering excipients that enhance tissue uptake while supporting vascular healing. The variability in clinical outcomes across paclitaxel-, sirolimus-, and biolimus-based DCBs reflects the importance of this interplay and reinforces the need for mechanistic research, computational modeling, and standardized endpoint evaluation in future trials [33].

In parallel, the emerging field of computational modeling has opened new avenues to optimize DCB technologies and refine clinical use. Computational simulations—including computational fluid dynamics (CFDs), pharmacokinetic transport models, and finite element analyses—are increasingly employed to predict how drug concentration, coating composition, inflation time, and arterial wall properties influence drug delivery and retention. These models offer valuable insights into the mechanistic underpinnings of DCB efficacy, often allowing for in silico optimization prior to costly clinical development. For instance, studies have shown that excipient properties and drug release kinetics can be mathematically tuned to optimize tissue uptake under realistic flow and wall conditions [34]. Moreover, patient-specific coronary artery models have been developed to simulate drug distribution based on individualized anatomy and hemodynamics, providing the foundation for personalized DCB treatment strategies [35]. These computational approaches not only guide the next generation of DCB development but may also enhance procedural planning by helping operators identify optimal balloon sizing, inflation protocols, and lesion selection strategies. As these models become more integrated with clinical imaging and physiologic assessment tools, they hold significant promise for improving both device design and procedural outcomes. Ultimately, computational modeling represents a transformative tool to enhance precision in interventional cardiology, enabling more individualized and evidence-based deployment of DCB technology.

## 7. Conclusions

The recent advancements in DCB and DES technologies underscore the evolving landscape of percutaneous coronary intervention. These studies have provided valuable insights into the comparative safety and efficacy of these approaches across diverse patient populations and lesion complexities. DCBs demonstrate promise as viable alternatives to DESs in different settings of the spectrum of CAD, particularly in small vessel disease, where the current era of DESs can be challenging and is sometimes outperformed by DCBs. Emerging strategies and technology advancements, such as blended PCI (DES + DCB in long lesions) and new drug formulations, could address challenges in complex CAD by reducing stent-related complications and promoting vascular restoration. A growing body of evidence suggests that the combination of drug properties and excipient formulation critically determines the consistency and durability of DCB efficacy. In parallel, computational modeling has emerged as a powerful tool to simulate drug delivery, optimize excipient performance, and guide individualized DCB therapy planning. While these findings highlight the great potential of DCBs in competing with DESs, it is crucial to reduce the variability in clinical and angiographic/physiological endpoints across trials in order to permit comparisons across studies and decrease the possibility of wasting potential new technologies in the field.

## Figures and Tables

**Table 1:** DCB devices commercially available for use in coronary artery disease.

Device	Company	Excipient Technology	Drug Concentration (µg/mm^2^)	Certification
Paclitaxel-Coated Balloons				
Agent	Boston Scientific	ATBC	2	CE, FDA
Biostream	Biosensors	Shellac	3	CE
Danubio	Minvasys	BTHC	2.5	CE
Dior II	Eurocor	Shellac	3	CE
Elutax SV	Aachen Resonance	Dextran Sulfate	2.2	CE
Essential Pro	iVascular	BTHC	3	CE
Prevail	Medtronic	Urea	3.5	CE
Pantera Lux	Biotronik	BTHC	3	CE
Restore	Cardionovum	Shellac	3	CE
SeQuent Please	BBraun	Iopromide	3	CE
Sirolimus-Coated Balloons				
MagicTouch	Concept	Nanocarriers	1.27	CE
Mozec SEB	Meril	Solid Lipid Nanospheres	3	CE
Selution	Cordis	Biodegradable Polymer as well as nanocarriers	1	CE
SeQuent SCB	BBraun	BHT	4	CE

Abbreviations: ATBC: acetyl tributyl citrate; BTHC: n-butyryl tri-n-hexyl citrate; BHT: butylated hydroxytoluene; CE: Conformité Européenne certification; FDA: US Food and Drug Administration approval.

**Table 2:** Key summary of recent randomized controlled trials or MA, evaluating DCBs for the treatment of coronary artery disease.

Trial Name	Study Type	N	Population	Interventions	Primary Endpoint	Results	Conclusion	Country of Study
REC-CAGEFREE I	Non-Inferiority	2272	Patients with de novo, non-complex coronary lesions; low/moderate CV risk	Swide DCB vs. Firebird 2 DES	Device-oriented composite endpoint (DoCE) at 2 years follow-up	Primary endpoint: DCB 6.4%, DES 3.4% (*p*-non-inferiority = 0.65).	DCB Failed to prove non-inferiority	China
Dissolve SVD	Non-Inferiority	277	Single lesion de novo SVD-CAD; low/moderate CV risk	Dissolve DCB vs. Resolute Onyx DES	In-segment diameter stenosis at 9 months	In-segment diameter stenosis: 31.2% ± 2.0% (DCB) vs. 26.1% ± 2.1% (DES), [*p*-non-inferiority = 0.0002]	Non-inferiority of DCB to DES was proven	China
ANDROMEDA	Meta-analysis	1475	De-novo SVD CAD	PCB vs. DES	MACE at 3 years	(DES) 18.5% vs. 24.5% (DCB); HR 0.67, 95% CI 0.47–0.96, *p* = 0.027	PCB is associated with a lower risk of MACE than DES	Multi-country
TRANSFORM I	Non-Inferiority	121	Stabilized acute or chronic coronary syndromes; small vessel lesions (mean RVD: 2.05 mm)	MagicTouch SCB vs. SeQuent Please Neo PCB	Angiographic net lumen gain at 6 months	Net lumen gain: 0.25 ± 0.40 mm (SCB) vs. 0.48 ± 0.37 mm (PCB), *p*-non-inferiority = 0.173 (SCB failed non-inferiority).	SCB failed to show non-inferiority	Italy, Ireland, United Kingdom
SeQuent SCB vs. SeQuent PCB	Non-Inferiority	70	Stable and unstable angina; de novo stenosis (mean vessel size: 2.95 mm)	SeQuent SCB vs. SeQuent PCB	LLL at 6 months	In-segment LLL: 0.11 ± 0.37 mm (SCB) vs. 0.04 ± 0.39 mm (PCB). Non-inferior (mean difference: 0.07 mm; *p*-non-inferiority < 0.001).	SCB demonstrated non-inferiority to PCB	Germany
ISAR-DESIRE 3 (7 yr)	Superiority	262	Patients with DES-ISR	Agent PCB, SeQuent Please PCB	TLR at 7 years	TLR: 43.2% (Agent PCB) vs. 35.9% (SeQuent PCB), *p* = 0.205.	No significant difference for the primary endpoint.	Germany
ISAR-DESIRE 3 (10 yr)	Superiority	402	Patients with DES-ISR; 10 years follow-up	PCB vs. PES vs. PB	Composite of cardiac death, target vessel MI, TLT, TLR at 10-year	Composite endpoint: 55.9% (PCB), 62.4% (PES), 72.0% (PB), *p* < 0.001.	PCB and PES superior to PB.	Germany
BIO ASCEND ISR	Non-Inferiority	280	Patients with DES-ISR across 17 centers	BCB vs. SeQuent Please NEO PCB	LLL at 9 months	LLL: 0.23 ± 0.37 mm (BCB) vs. 0.25 ± 0.35 mm (PCB), *p*-non-inferiority < 0.0001.	BCB demonstrated non-inferiority to PCB	China
AGENT IDE	Superiority	600	Patients with ISR (reference vessel diameters 2.0–4.0 mm)	Agent PCB vs. Uncoated Balloon	1-year TLF	TLF: 17.9% (PCB) vs. 28.6% (Uncoated Balloon), *p* = 0.003. PCB superior.	DCB is superior to uncoated balloon	USA
DCB-BIF	Superiority	784	Patients with true coronary bifurcation lesions; side branch treatment	SeQuent Please NEO PCB vs. NCB	MACE at 1 year	MACE: 7.2% (DCB) vs. 12.5% (NCB), *p* = 0.013. DCB reduced MACE, particularly MI (1.0% vs. 3.6%, *p* = 0.029).	DCB is superior to NCB	Primarily China (with Italy/Germany minor participation)
ULTIMATE III	Superiority	260	High bleeding risk patients with de novo coronary lesions	IVUS- vs. Angiography-Guided (SeQuent Please PCB)	In-segment LLL at 7 months	LLL: −0.10 ± 0.34 mm (IVUS-guided) vs. 0.03 ± 0.52 mm (Angiography-guided), *p* = 0.025. Lower LLL with IVUS.	IVUS-guided DCB superior to angiography-guided DCB	China
PICCOLETO VI	Superiority	297	Patients with de novo small-vessel coronary artery disease	Multiple PCBs vs. SCBs	Late Functional Loss at 6 months	Functional loss: −0.01 ± 0.15 (PCB) vs. +0.03 ± 0.13 (SCB), *p* = 0.09.	No significant difference in late functional loss across groups	Italy
REFORM	Non-Inferiority	202	Patients with DES ISR	BCB vs. SeQuent Please DCB	In-segment percentage diameter stenosis at 6 months	In-segment percentage diameter stenosis: 43.3% ± 22.9% vs. 31.4% ± 17.7%) (*p* non-inferiority = 0.48)	BCB failed to show non-inferiority	France, Germany, Italy

Abbreviations: DoCE (device-oriented composite endpoint); DCB (drug-coated balloon); DES (drug-eluting stent); SCB (sirolimus-coated balloon); PCB (paclitaxel-coated balloon); BCB (biolimus A9-coated balloon); TLF (target lesion failure); LLL (late lumen loss); MACE (major adverse cardiac event); MI (myocardial infarction); TLR (target lesion revascularization); IVUS (intravascular ultrasound); PB (plain balloon); PES (paclitaxel-eluting stent); NCB (noncompliant balloon); RVD (reference vessel diameter). Statistical significance is indicated by *p*-values, and *p*-non-inferiority values specifically apply to non-inferiority trials.

**Table 3:** Key ongoing clinical trials of DCB for the treatment of coronary artery disease.

ClinicalTrials.gov Identifier	Study Title	Country	Study Design	Intervention	Control	Conditions	Enrollment	Primary Endpoint	Follow up
NCT04280029	SELUTION 4 ISR trial	United States of America, Belgium, Brazil, Canada, France, Italy, Netherlands	Multicenter, single blind, non-inferiority	SELUTION SCB (Cordis)	POBA or DES	ISR	418	TLF at 1 year	Up to 1 year
NCT04814212	DEBATE trial	Finland, Germany, United Kingdom	Multicenter, double blind, non-inferiority	PCB	DES	HBR	546	MACE and BARC 2-5 at 1 year	Up to 36 months
NCT04859985	SELUTION DeNovo study	Austria, Czechia, Finland, France, Germany, Italy, Netherlands, Poland, Singapore, Spain, Switzerland, United Kingdom	Multicenter, single-blind	SELUTION SCB	DES	De novo CAD in large vessels	3326	TVF at 1 and 5 years	Up to 5 years
NCT04881812	Co-CTO trial	Netherlands	Single-center, single-blind, non-inferiority	DCB	DES	CTO	144	%DS at 1 year	Up to 1 year
NCT04893291	TRANSFORM II trial	Italy, Bangladesh, Netherlands, France, Spain	Multicenter, open-label, non-inferiority	MagicTouch SCB (Concept Medical)	DES (everolimus)	SVD	1820	TLF and NACE at 1 year	Up to 5 years
NCT04896177	Sirolimus DEB in bifurcation lesions	China	Multicenter, open-label non-inferiority,	SCB (Shenzhen Salubris Pharmaceuticals)	PCB (Liaoning Yinyi Biotechnology)	Bifurcation	280	%DS of target lesion branch at 9 months	Up to 2 years
NCT04918615	PROMISE-BIF trial	China	Multicenter, open-label, non-inferiority	SCB (Shanghai MicroPort Medical Group)	PCB (Liaoning Yinyi Biotechnology)	Bifurcation	236	%DS of side branch at 9 months	Up to 2 years
NCT05209412	CAGE-FREE III trial	China	Multicenter, open-label, non-inferiority	PCB (Lepu Medical Technology)	DES	De novo CAD in large vessels	370	FFR at 1 year	Up to 1 year
NCT05221931	DCB-HBR trial	Republic of Korea	Multicenter, open-label, non-inferiority	Agent PCB (Boston Scientific), Prevail PCB (Medtronic), or SeQuent Please PCB (B-Braun)	DES	HBR	1350	TVF at 2 years	Up to 2 years
NCT05544864	ISAR-DESIRE 5 trial	Germany, Spain	Multicenter, open-label	DCB	DES	ISR	376	MACE at 2 years	Up to 2 years
NCT05680051	DCB Under the Guidance of OCT in STEMI	China	Multicenter, open-label	DCB (Lepu Medical Technology)	DES	STEMI	300	LLL at 10 months	Up to 10 months
NCT05731687	Hybrid-DEB trial	Netherlands	Multicenter, single-blind, non-inferiority	MagicTouch SCB	DES	Bifurcation	500	MACE at 2 years	Up to 2 years
NCT05750771	DES vs. DCB in calcified de novo lesions	China	Single-center, open-label, non-inferiority	PCB	DES	De novo calcified lesions in elderly	200	LLL at 1 year	Up to 1 year
NCT05846893	REVERSE trial	Korea, Taiwan, Malaysia, Singapore	Multicenter, open-label, non-inferiority	SeQuent Please PCB	DES	De novo CAD in large vessels	1436	NACE at 1 year	Up to 36 months
NCT05908331	MAGICAL ISR trial	United States of America	Multi-center, single-blind	SCB MagicTouch	POBA	ISR	492	TLF at 1 year	Up to 5 years
NCT05946629	SELUTION 4 IDE trial	United States of America	Multi-center, single-blind	SELUTION SCB	DES	SVD	960	TLF at 1 year	Up to 5 years
NCT06365502	RESTORE trial	China	Multi-center, open-label	PCB	GDMT	Non-flow limited vulnerable plaque in ACS	1860	TLF at 2 years	Up to 2 years
NCT06448637	DEBORA study	Spain	Multi-center, open-label	DCB	DES	De novo CAD in large vessels	94	Positive response vasomotor function at 8 months	Up to 8 months
NCT06746233	BOOST-AMI trial	Serbia	Multicenter, open-label	PCB	DES	STEMI	598	DoCE at 1 and 2 years	Up to 2 years

Abbreviations. %Ds: percent diameter stenosis; ACS: acute coronary syndrome; STEMI: ST Elevation Myocardial Infarction; BARC: bleeding academic research consortium; CTO: chronic total occlusion; DCB: drug coated balloon; DES: drug-eluting stent; DoCE: device-oriented composite endpoint; FFR: fractional flow reserve; GDMT: guideline-directed medical therapy; HBR: high bleeding risk; ISR: in-stent restenosis; LLL: late lumen loss; MACE: major adverse cardiac event; NACE: net adverse cardiac event; MLD: minimal luminal diameter; PCB: paclitaxel-coated balloon; POBA: plain balloon angioplasty; SCB: sirolimus-coated balloon; SVD: small vessel disease; TLF: target lesion failure; TVF: target vessel failure.
